# Health promotion at local level: a case study of content, organization and development in four Swedish municipalities

**DOI:** 10.1186/1471-2458-10-455

**Published:** 2010-08-03

**Authors:** Elisabeth VG Jansson, Per E Tillgren

**Affiliations:** 1 School of Health, Care and Social Welfare, Mälardalen University, Box 883, SE-721 23 Västerås, Sweden; 2 Department of Public Health Sciences, Karolinska Institutet, Norrbacka, SE-171 76 Stockholm, Sweden

## Abstract

**Background:**

Several health determinants are related to local conditions and prerequisites at community level. For this reason, strengthening community action has been one of five strategies implemented in health promotion since the end of the 1980s. Such action includes setting priorities, making decisions, planning strategies, and implementing them to achieve better health. The aim of this paper is to obtain a deeper understanding of content, organization and processes in the development of local health promotion.

**Methods:**

A qualitative multiple case study of four Swedish municipalities. The cases were analyzed in accordance with the principles of cross-case study analysis, and a content analysis of documents and interviews was conducted in two steps. First, a manifest content analysis was performed to identify present and former actors and measures. Thereafter, a latent content analysis was performed to investigate structures and processes in local contexts.

**Results:**

The results of the inductive content analysis showed development of local health promotion in three phases: initiation, action, and achievement. Strengthening factors were local actors, health statistics and events. Hindering factors were lack of resources and vague objectives. External factors, e.g. national policies, were not perceived as prominent influencing factors. Media reports were regarded as having had an influence, but only to some extent. The content of local health promotion has developed from ad-hoc lifestyle and behaviour-related actions into structural, intersectoral actions related to determinants of health.

**Conclusions:**

The municipalities have organized and developed their health promotion targets, actions and priorities on the basis of local needs and prerequisites. The three phases in the identified health promotion processes were experienced and documented as being subject to greater influence from internal rather than external strengthening and hindering factors in their local contexts.

## Background

The importance of local health promotion has been emphasized in several WHO documents since the 1970s. The Ottawa Charter, for example, advocated strengthening community involvement as one strategy to achieve equity and better health, involving actions such as setting priorities, decision-making, planning and the implementation of health-promotion strategies [1]. The idea of the effectiveness of a community approach to health promotion is attractive, since several factors known to impact on people's health are related to social structures and community environments [2-5].

A community's social structures provide opportunities to reach out through already existing connections and organizations in a local environment to which people more easily can relate. Such bonding enhances participation and engagement in community development [5,6]. The recent practice of health promotion has emerged from a disease and risk prevention perspective, but has developed towards an approach based on health determinants [7]. Addressing health determinants that can be related to social interventions has been shown to be more efficient than taking individually directed actions [8]. Nutbeam and Harris [3] define community organization in terms of processes through which community groups are assisted in identifying common problems or goals, mobilizing resources, and in other ways developing and implementing strategies for reaching their collective goals. When communities enhance their ability to identify, mobilize and deal with health problems, community capacity is strengthened.

There is a diversity in health promotion, where disease and injury prevention is often organized in top-down programmes, driven by external actors in partnership with communities [9]. Community development, on the other hand, focuses on building community capacity, and is often directed at determinants of health. In its ideal form, community development is founded in a bottom-up approach and arises at grassroots level in the community.

Local health promotion is influenced and dependent on external factors, such as national and international decisions and policies [10]. Moreover, any community or municipality with its own goals, needs and prerequisites exists within a system of individuals, structures and relations [2,11]. And, from a municipal organizational perspective, it is implied by social change theories that changes at community level require changes in the whole system - from individuals, via subsystems and alliances, to the external environment - and the interrelationships between all system components [11].

In terms of policy formulation and development, Kingdon [12] has described a dynamic process for agenda setting that has three independent streams: problem, politics, and policy. In some situations, the three streams merge with one another, which provides opportunities for a policy change. The Kingdon model was initially developed for policy processes at national level, but has also been applied at local level in matters related to public health [13-15].

The processes involved in health promotion are characterized by and dependent on components like social planning and action, local ownership and communities' opportunities to adapt to their own local needs and resources [1,2,11,16,17]. Increased capacity building enhances opportunities operationally to develop more effective and long-term health promotion in the community [18].

In Sweden, there are three politically elected levels of government: national, regional and local. The development of health promotion started at regional level in the health and medical sector of the Swedish counties' in the 1970s, and was followed by involvement at local level (in municipalities) at the end of the 1980s [19].

In 2003, the first national public health policy (PHP) was approved by the Swedish parliament. Its overall aim is 'to create societal conditions for good health on equal terms for the entire population'. The PHP contains eleven domains of objectives linked to policy sectors that affect three groups of determinants of health: structural factors, living conditions, and lifestyles [20]. A key role for municipalities is expressed in the PHP, which emphasizes the value of developing intersectoral health promotion with explicit goals and strategies. There is, however, no national funding for intersectoral health promotion within the confines of the PHP.

At local level, Swedish municipalities possess institutional self-governance and independence within a legislative framework determined by the Swedish parliament [21]. The municipalities have taxation rights, and have responsibilities in several public-service areas, e.g. care for the elderly and children, local planning and building, compulsory schooling, and cultural and leisure-time facilities, but there are no explicit rules or statutes covering health-promotion plans or actions. On the other hand, there is legislation that concerns specific health risks; for example, the municipalities are the authorities responsible for local supervision of the provisions of the Tobacco Act.

Studies of municipalities as dynamic and comprehensive systems can provide knowledge of the use of resources and actions in these self-governed government units. The aim of this paper is to obtain a deeper understanding of the content, organization and processes in the development of local health promotion from the 1980s through to 2006. What health promotion activities are there in the municipalities? What are their content and priorities? How were they organized and by whom? Finally, how and why were the processes developed?

## Methods

This is a retrospective multiple case study [22], in which an exploratory and inductive approach, from a municipal perspective, was adopted. The study design is intended to capture municipalities' unprejudiced views on their own work with local health promotion.

Case studies concentrate on defined and autonomous cases in order to obtain in-depth understanding of specific contextual conditions. They also offer a systematic way of collecting, handling and analyzing an extensive quantity of data in each single case [23]. The four cases were purposefully selected, based on a community analysis in accordance with Bracht and colleagues' Five-Stage Community Organization Model for Health Promotion [24,25]. The intention of the selection was to obtain typical cases from a group of municipalities in a similar geographical area, but with internal differences in aspects such as political leadership and governance.

The cases are municipalities located in two Swedish counties that include the south and west of Lake Mälaren, each with a population of between 10 and 30 thousand (see Table 1). The municipalities themselves are major employers in the delivery and maintenance of local public services. There is though a divergence between the municipalities with regard to the structure of industry, political leadership and municipal governing organization. Local trade and industry differ between the municipalities, but include both small/medium-sized enterprises and large manufacturing companies. The political leadership varies; there are both socialist and non-socialist majorities, and also different kinds of coalitions.

**Table 1:** Municipality characteristics in 2006 [40,41]

	*Demographics*	*Gainfully employed, employers, trade and industry*	*Local government 1980-2006*
**Municipality A** (Oxelösund) Land area 36 km^2^	11,000 inhabitants (310/km^2^) A sixth foreign born Average length of life ♀ 82 years, ♂ 77	1,900 lived and worked in the municipality The municipality employed a fifth, a single manufacturing industry half, with the remainder in docks, public services, private companies, etc. 800 outward commuters	Mainly a Social Democratic Party majority

**Municipality B** (Strängnäs) Land area 980 km^2 (2007)^	30,200 inhabitants (40/km^2^) A tenth foreign born Average length of life ♀ 82 years, ♂ 78	4,100 lived and worked in the municipality The municipality employed a forth, small and medium-sized enterprises in manufacturing and pharmaceuticals a fifth, with the remainder in private and public service companies, etc. 3,600 outward commuters	Coalition of mainly the Conservative and Centre Party

**Municipality C** (Fagersta) Land area 312 km^2^	12,300 inhabitants (45/km^2^) A fifth foreign born Average length of life ♀ 86 years, ♂ 76	2,400 lived and worked in the municipality The municipality employed less than a fifth, engineering and manufacturing industries a half, with the remainder in private and public service companies 500 outward commuters	Social Democratic Party dominance in the 1980s. Since 1998 a Left Party majority

**Municipality D** (Sala) Land area 1,211 km^2^	21,700 inhabitants (20/km^2^) A tenth foreign born Average length of life ♀ 82 years, ♂ 79	3,100 lived and worked in the municipality The municipality employed a third, small and middle-sized trade and manufacturing enterprises a sixth, with the remainder in private and public service companies, and in forestry and agriculture 2,100 outward commuters	Mainly a coalition of the Centre and Conservative parties. Not always a clear majority for the coalition

Data comprised municipal strategic documents and interviews. The documents consisted of local policies, plans, minutes from the 1980s through to 2006, and annual reports for the period 1998 to 2006.

A sample of key informants with knowledge of each municipality's local activities and intentions was purposefully selected. Semi-structured interviews with 30 officials and six politicians were performed between December 2005 and September 2006. The interview guide contained questions based on a framework of guiding principles for health promotion - empowering, participatory, holistic, intersectoral, equitable, sustainable, and multistrategic [26], all with a focus on local organization and development. The electronically recorded interviews lasted 70 minutes on average, and were conducted and transcribed by the first author.

The cases were analyzed in accordance with principles for case study analysis. The general analytic strategy was to perform a time-series analysis, built on 'How' and 'Why' questions about relationships and changing events over time [22]. The four municipalities were initially treated as individual 'vertical' cases, and were analyzed one by one. Thereafter, they were compared 'horizontally' in a cross-case analysis.

The collected data, documents and interviews in each case, were analyzed inductively, following the principles of manifest and latent content analysis, which involved the identification of categories and themes [27]. The interview guide and analytic procedure were initially piloted in a different but similar municipality. Inductive analysis entails that patterns, themes and categories emerge from the data rather than being imposed on the basis of previously performed data collections and processing [23].

The content analysis was conducted in two steps. Step one consisted of a retrospective manifest content analysis, with a focus on content, organization and important events, elements that are known to have had a notable impact on local health-promotion development. Step two consisted in a latent content analysis of the underlying processes and structures that influenced the development of local health promotion.

The research project was approved by the regional ethics committee. The four municipal administration boards gave their support for participation, and all respondents gave verbal informed consent.

## Results

The findings will be presented as follows: first, the content and organization of local health-promotion activities will be described; second, the results of an analysis of factors influencing developments within the four municipalities will be presented.

### Local health promotion's content, organization and development

What the four municipalities had in common in the 1980s was a lack of any formal organization or explicit strategy for health promotion. The expected outcomes and obtained effects of different individual and disease-prevention measures taken in the 1980s and at the beginning of the 1990s focused on reducing the incidence of diseases, e.g. cancer or cardiovascular disorders. Actions were usually taken ad-hoc and performed by single departments, and mainly concentrated on changing individuals' patterns of behaviour.

Inter-municipality differences were related to municipal characteristics that influenced strategies and actions (see Table 2). Municipality A had several prevention projects, one concerning alcohol, in collaboration with the single largest local company employer. Municipality B had a general focus on children and youth, and in 1998 child assessments were made to obtain basic information for decisions and actions. In Municipality C, problems related to companies closing down and people moving out of a strained economic situation prompted the development of several forms of intersectoral internal and external collaboration, such as a family centre, which was set up in 2004. In Municipality D, the municipal profile was related to environmental and ecological issues. In this context, a health-planning officer, positioned in and funded by the county council, was a driving force in the initial use of an assessment tool within a local welfare management system in 2000.

**Table 2:** Municipal organization of local health promotion in 2006

		***Case A***	***Case B***	***Case C***	***Case D***
**Organization**	*Municipal administration organization*	Centralized administration management with administrative offices	Centralized administration management with administrative offices	Decentralized administration management	Decentralized administration management

	*Expressed municipal administration responsible for local HP issues*	Municipal executive committee	Unclear, unexpressed	Municipal executive committee	Culture and leisure department

	*Local public health committee/Chairman*	Yes/The chair of the Social Services Committee	No	Yes/The leading councillor	No

	*Local health-planning officer*	No	No	Yes, county financed with location in the municipality	Yes, county financed with location in the municipality

	*External actors and partners*	NGOs, public authorities. local trade and industry, neighbouring municipality	NGOs, public authorities, local trade and industry	County, NGOs, public authorities, local trade and industry, neighbouring municipalities	County, NGOs, public authorities, local trade and industry

**Management**	*Municipal vision*	Pride and belief in the future	Child and youth municipality	Life time in Fagersta	Ecological municipality

	*Main municipal administrative goal*	Improved community environment. More housing. Increased participation. Increased public health. Follow-up of the local disability programme	Long-term sustainable economic development with an annual increasing net cost	A developing and safe environment that enables people and companies to be active and grow	Increasing growth through the utilization of companies' development opportunities and the municipality's geographical location to develop and ensure common welfare

	*Intermediate goals related to health promotion*	Decreased sick leave. Increased access to physical activity	A complete society. Increased participation	More meeting places. Safe city environment	Sustainable development. Safe and secure municipality

	*HP plans, policies and programmes*	Alcohol and drug programme	Political programmes for alcohol- and drugs, culture and leisure. Family centre	Programme for alcohol and drugs. Action plan for children at risk of harm. Family centre. Occupational programme for youth	Alcohol and drug programme. Labour-market policy programme. Rehabilitation policy for municipal employees

	*Follow-up and evaluation of health promotion activities*	Equality assessment. Some questionnaires. Some evaluation	Child assessments. Equality assessments. Several questionnaires. Some evaluation	Equality assessment. Some questionnaires. Some evaluation	Equality assessment. Some questionnaires. Some evaluation. Local welfare management system

**Focus**	*Target group*	Children, youth, municipal employees, local-company employees	Children, youth, elderly, municipal employees	Children, youth, elderly	Children, youth, municipal employees

	*Target areas*	Alcohol, tobacco, physical activity	Alcohol, drugs, participation, physical activity	Alcohol, drugs, physical activity, safe environment	Alcohol, drugs, physical activity

	*Strategies*	Internal and external intersectoral collaboration. Projects and programmes	Intersectoral collaboration. Projects and programmes	Networks. Internal and external intersectoral collaboration. Collaboration between municipalities. Projects and programmes	Intersectoral collaboration. Projects and programmes

Alcohol prevention has been the single most prioritized area for local health promotion in all four municipalities since the 1980s. In terms of age, the most prioritized target group over time in all municipalities was children and youth, although in recent years greater attention has been paid to the elderly and municipal employees.

From the mid-1990s onwards, health-related objectives were formalized and realized at municipal-department level, particularly in municipalities A and D. The municipalities' administrations started to build up a more goal-oriented and structured organization for health promotion. In these two municipalities, there was an interest and willingness on the part of two heads of department to function as "champions" of public health, which was a strong predictor of action being taken. They both advocated a more holistic view on local health promotion and intersectoral cooperation.

At the end of the 1990s, more overall municipal goals in varying public-health areas came to be formalized and realized, e.g. alcohol and drug policies. Towards the end of the century, a diverse range of external and internal intersectoral collaborations in local health promotion was established. For example, there were the intersectorally composed public-health committees, with representatives from both inside and outside the municipal organization, that were set up around 1995 in municipalities A and C. The role of the committees has changed from being notionally operative at the beginning to becoming a forum for information and knowledge exchange. In the mid-1990s, Municipality B began to focus on children and youth, and implemented child impact analysis as a tool in municipal planning and decision-making. The municipality's overall focus on children has served as a framework for several internal and external intersectoral health-promotion activities. In Municipality D, Agenda 21 and environmental issues became the over-riding area of concern; although, from a political point of view, this was expressed as a framework for local health promotion, the implications of this view were not fully implemented at departmental level.

Around 2002, there was a shift towards giving local health promotion a place in municipal policies. But the causes or reasons for this remain unclear. In the first couple of years of the new millennium, expressions of the expectation that municipalities would operate in relation to the determinants of health referred to in the forthcoming national PHP became more common. Formulations such as better health, increased participation, a safe and secure childhood, adolescence and old age, and also a safe workplace, started to appear in the municipalities' visions and goals. Although there were still no explicit objectives or visions with regard to increased equity in health, several kinds of actions and strategies in accordance with the PHP objective domains were implemented. The municipalities also declared increased participation to be an important objective, but there were no strategies for how to obtain it (see Table 2).

A common trend in the municipalities was that local health promotion changed from ad-hoc activities in decentralized and sector-oriented organizations in the 1980s to more intersectoral, goal- and society-oriented activities in the 1990s. This can be exemplified by citing one official from Municipality A:

"Fifteen years ago, we had a lot of departments organized in pipelines. Then, we had the client-contractor model, where everyone was focused solely on their own business in separate result units. Today, we have fewer committees and a process of breaking down walls and increasing cooperation" (Municipality A, 9 years as head of department).

Or, as a politician expressed it:

"Formerly, it [health promotion] was just the work of local 'champions' in small units who worked on individual projects; you just wheeled out the cannon, took one shot, and that was it" (Municipality A, 25 years as a politician).

### The local health promotion development process

Local health-promotion development in the four municipalities has been influenced by both explicitly expressed and unexpressed factors in both planned and unplanned processes. From the content analysis, three central categories emerged, which also represent three phases in a development process: initiation, action, and achievement (see Table 3).

**Table 3:** Processes in the development of local health promotion

*Theme*	The content and development processes in local health promotion influenced by internal and external contextual factors, which emerge both intentionally and unintentionally						
*Categories*	Initiation		Action		Achievement		

*Sub-categories*	Origin	Catalyst	Support	Measure	Structure	Strategy	Expected outcome

#### Initiation

The initiation phase in the four municipalities had its origin in various internal and external context-influencing factors. The internal factors were local issues and needs with a long history in the municipality in question, which were related to local politics, economy or demography. The external factors were principally national recommendations and objectives, and to some extent health trends in society.

The originating factors then required one or more catalytic factors for the issues to be practically addressed. The most evident internal catalysts were the presence of a local champion or enthusiast and important local events, while the most evident external catalysts were funding, statistics on the locality, and media reports. One official expressed the view that action was predominantly event-related:

"The basis for action, I believe, lies in events in the community, or the different kinds of impacts that gets someone to work on some issue [...] the everyday ongoing policy or whatever you want to call it. So, it is of course very, very event-related" (Municipality A, official for 19 years).

Important local events have impacted on the communities and their political agendas, and have generated several health-promoting objectives and actions. In municipalities A and D, for example, there were incidents of alcohol-related fatal assaults on young men. These events attracted considerable local attention, and contributed to intersectoral mobilization for alcohol-preventive action, which also received external aid and funding. Local public-health statistics, as they appear in media or county-council reports, also act as catalysts.

There were "champions" of health promotion both within the municipal departments and in external sectors, such as branches of the local welfare and employment agencies or NGOs. Core members of the municipalities, and in particular champions or enthusiasts at different municipal levels, have also had an impact on all phases of the development process.

Another example lies in the heads of departments in municipalities A and D, who have advocated and established health-promotion objectives and actions in their own departments since the 1990s. They have also tried to implement these ideas throughout their municipalities' administrations.

Health promotion has not historically been a prioritized issue on local political agendas. But a change could be observed around 2002, when politicians began to discuss health-promotion objectives and actions more frequently, especially in municipalities A and B. A politician in Municipality B described this as an effect of growing interest and action in the community:

"... the engagement must, of course, be to someone or be related to some special event, or some association might take the initiative. In my work, I can see that much of the input comes from associations, on different issues. [...] And I believe [...] a lot in this, because then the commitment comes from several people or organizations" (Municipality B, politician for 9 years).

External international and national health-related policies have to some extent acted as a wakeup call, primarily by initiating new decisions and actions at departmental level. This was most evident when the actions corresponded with a champion's and/or a municipality's own area of interests. In 1996, the United Nations Convention on the Rights of the Child supported politicians in Municipality B with regard to their expressed profile on children and youth. Agenda 21 worked as a foundation for Municipality D's profile on the environment. Public health policies, such as the WHO Regional Office's Health for All targets from 1993, and the preparatory work of the Swedish PHP, conducted by the National Committee for Public Health during the period 1997-2000 had by 2002 led to the setting-up of administrative targets in municipalities A and D. This entailed documented actions and the formulation of internal health-promotion objectives. By 2003, the Swedish PHP was also being implemented at executive level in Municipality A.

At regional level, the county councils have been an external originating and catalytic factor, and an initiator and operator in the municipalities' local health promotion. The county councils have influenced activities through the provision of financial and individual aid, and the dissemination of knowledge. In among other ways, this has come about in the form of targeted project funding with the aim of stimulating the development of health promotion at local level by providing expertise or appointing locally placed health-planning officers.

#### Action

The second phase, the action phase, entails that planned or emerging public-health issues are formalized and addressed in various decisions or measures. The action phase has also been influenced by internal and external hindrances and strengthening factors in relation to and during health-promotion activities.

Around 2002, in Municipality A, there was the initiation of a process of systematically working towards some all-embracing, municipality-wide objectives, adapted to local needs and resources. This work was supported at the highest administrative and political level in the municipality. Two core members, the administrative chief executive officer and the political leading councillor, worked together to implement these new objectives throughout the municipal organization. One of the new goals for 2006 was better public health. The goals obtained considerable support at all levels within the municipal organization, and were also referred to in contacts with external collaborating partners. Perceived positive effects of the new municipality-wide goals were that they facilitated the setting of priorities and choice of measures; also, it was regarded as easier to coordinate intersectoral health-promoting interventions when everyone had the same focused target to work towards.

In Municipality B, as in the other municipalities, children and youth became the most prioritized target group. Support for these issues in Municipality B was also formulated and rooted politically, which has led to decisions on municipality-wide activities. Between 1996 and 1998, this led to a decision that so-called child-impact analyses should be performed prior to all municipal operational decisions, and also that activities should be followed up annually with child accounts prepared in accordance with the provisions of the United Nations Convention on the Rights of the Child. However, up until 2006, there was no substantial explicit support for or focus on public-health issues at municipal or administrative level.

Nor in Municipality C was there any comprehensive municipality-wide orientation towards health promotion. However, proximity to and clarity on the part of municipal management was regarded, at administrative level, as having given explicit support for various health-promotion measures, and also as having facilitated collaborative intersectoral implementation. Also, in conjunction with the new general municipality objectives being adopted in 2002, funding was allocated, for 2003, in a separate budget. There, municipal departments could seek financial support for various health-promoting projects or interventions.

However, Municipality D, which had a clearly expressed community-wide environmental profile, proved to receive only limited support for its approach in its municipal departments. At administrative level, however, there were within one of the departments both explicit targets for public health, and several measures focusing on prevention and health promotion had been implemented. An externally introduced pilot project involving the preparation of a local welfare management system - a follow-up tool covering health indicators in year-end accounts - was started in 1998 in collaboration with the county council's health-planning officer and other external actors. A political decision was taken in 2000 to continue work on welfare accounting, which meant that year-end accounts were also prepared for 2001 and 2004. But, since a political decision was lacking on how the accounts should be used, the accounting results never obtained legitimacy or their envisaged function as a basis for decision-making and planning.

One obstacle that was described in relation to the development of local health-promotion activities in the municipalities was the conception that the issue of their responsibility had not been clarified. This was regarded as one of the reasons why the municipalities did not elevate clearly formulated public-health issues on the municipal agenda, and accordingly did not produce plans and policies for them. Or, as a chief executive officer in Municipality D expressed it:

"... so, there is a hesitation running consistently over who should take on responsibility for all local health-promotion activities. There is a conception, I believe, held by most local politicians in the current organization [...] that the county council has a role to play, that's the way it is. At the same time, the county council is trying to set priorities among its own tasks, and push out this particular responsibility on to others, largely the primary municipalities. So there is, indeed, a lack of clarity [...] Thus, the county council won't say explicitly that they haven't met their responsibility in this area, and nor are we prepared to say that we have it" (Municipality D, chief executive for 2 years).

The degree of support for and success of administration-wide actions in the municipal organizations is described as being dependent on how clear and well-rooted public-health-related objectives and measures are out in the operational arena. This was regarded, in turn, as being influenced by the form of governance adopted by the municipalities. In this context, one head of department in Municipality A stated that centralized goal-oriented direction and control could have advantages in relation to obtaining community support and implementing municipality-wide and coordinated health promotion:

"Increased clarity enables several departments to work in the same direction, and I think that before, when there were four different departments here, it can be imagined that they followed their own paths, at least to some extent; now, we can see the overall goals and proceed more at the same pace [...] thus, there is more strength and power behind what we do, it's all clearer" (Municipality A, head of department for 4 years).

The opposite view was that centralized direction and control in the municipalities hindered a department's capacity to take the initiative and act, since any measure first had to obtain central support.

There was a virtual consensus that awareness and explicit support for public-health issues had increased in the four municipalities since the 1980s, both within the municipal organization and among the population in general, especially when decisions and measures were related to and based on local conditions and needs.

#### Achievements

The third phase in the development process involved in local health promotion consists in the expected or achieved results that actions generate. These are achievements that contribute to the creation of durable change in the organization, structures and strategies of health-promotion activities. As a continuation, such activities might be expected to achieve established goals and visions for improved health in the municipalities. Just as at the two preceding phases, the structures and methods that came to be employed were not always the products of conscious strategic choices. Local internal and external resources and needs influenced the development and organization even during this phase.

In 1995, a decision was taken in Municipality A to set up a public-health committee. Organizationally, the committee was positioned in the department where one of the municipality's most prominent champions of health-promotion activities was active. This department was also the body initially given the responsibility for public-health issues in the municipality. The public-health committee, however, operated primarily as a forum for the exchange of information and knowledge. The municipality also collaborated intersectorally with various local actors, including large companies, on long-term alcohol prevention.

The organization of health promotion in Municipality B has consisted of various measures that have been non-explicitly integrated into regular municipal operations, i.e. health-promoting activities that are implemented both internally by different departments and also in collaboration with different actors in the municipality. The municipality's focus on increased influence for its residents, and especially for children and youth, led, among other things, to a political decision to establish a youth council in 1999. The aim of the council was to strengthen democratic participation among young people by providing increased opportunities for dialogue with local politicians and officials - something that municipal representatives regarded as an important health-promoting intervention. In 1998, a family centre was set up through collaboration between the county council's various healthcare institutions and the municipality's social services, for the purpose of establishing a location for coordinated intersectoral support for families with children.

Health promotion in Municipality C was also described as being non-explicitly integrated into regular municipal operations. There was no clear municipality-wide organization or definition of actually performed health-promotion activities; nor was there any explicit distribution of responsibilities.

A local public-health committee, with the county council's health-planning officer as a supportive and driving actor, was started in 1995. The initial intention was that the council should consist of people mandated to reach decisions that would enable them to pursue more operative health-promotion activities. However, the council has functioned more as a forum for information and knowledge exchange. In the municipality, different forms of collaboration on different interventions, both internal and inter-municipal, have developed. For example, within the municipal organization, several departments have established networks in order to support families with problems. Also, in 2005, the municipality - in conjunction with the county council - initiated an intersectorally driven family centre.

In Municipality D, prominence was given to collaboration with local associations, as one of the most important factors in working with health promotion. In the municipality, there was a long tradition of collaborating with associations, primarily for the purpose of increasing access to and interest in physical activity. In 2001, a several-year-long project in schools was embarked upon in conjunction with local associations.

Public-health issues in the municipality (D) have, by tradition, been promoted by an enthusiast in a managerial position within the Department of Culture and Recreation. In 2002, the department employed a development officer for health and cultural issues, with responsibility, inter alia, for health-promoting projects focusing on preschool children. The county council's health-planning officer, the health-and-culture development officer and a project manager were regarded by many to be the ones who advocated and organized public-health issues in the municipality.

In all the municipalities, various internal and external forms of collaboration have developed with regard to local health-promotion activities. The municipalities (A and C) that have had more explicit and well-rooted centralized municipality-wide goal formulations regarded this as having facilitated and reinforced the development of intersectoral collaboration. It was also something that was highlighted as a strengthening factor with regard to both internal and external interaction by executives in municipalities B and D.

## Discussion

The main finding of this study is that local health promotion has primarily been developed on the basis of municipalities' own needs and areas of interest. Important local events and champions or enthusiasts were perceived as being more powerful driving forces in this development than external factors, such as national or international public-health policies.

The Swedish municipalities administer local welfare activities with regard to infrastructure and social care. They also have a number of statutory obligations of relevance to people's health, not only in terms of social care but also in relation to risks referred to in national legislation, concerning tobacco, alcohol, and the living and working environment. But neither these obligations nor their possible meanings in municipal public health were referred to in the local interviews or documents. Reasons for this might lie in the risk-factor orientation of the national laws and the absence of connections with unified public health. Also, the municipalities lack any local statements or definitions of local health promotion and what it stands for, and may not have ascertained relationships between the public-health laws and their own local health promotion. However, municipalities are local producers and actors with responsibilities for several areas of importance with regard to health determinants, and therefore have the opportunity to exert a positive influence on their residents' health and living conditions [5,6,20]. Community-based interaction between local movements increases concern about the importance of addressing determinants of health in health-promotion programmes [17]. When actions are related to health determinants, they have been shown to be more efficient than those that are individually directed [8]. Several of the relevant factors are addressed in this study.

The four municipalities' health-promotion activities have changed from being individual and lifestyle based to being founded in a more holistic view on health. And the prioritized areas have changed from disease and risk prevention towards a structural perspective with a focus on the determinants of health. These trends reflect a parallel development in health promotion in Sweden since the 1980s, which mirrors the international trends described by Catford [7]. Municipalities are influenced by and dependent on external factors [10], but in this study such influence and dependency, with regard to local health promotion content, organization and development, were not explicitly expressed.

To implement sustainable and efficient local health promotion, the participation and active engagement of community leaders and organizations are essential [2,24]. In all the municipalities, there have been one or more committed and knowledgeable core community members, both officials and politicians, who - implicitly or explicitly - have functioned as advocates and driving forces in the initiation of local health promotion.

In particular, in municipalities A and D, politicians and officials have played a key role in the development process. And, the higher the position they have occupied, the greater the impact they have had. They have had the function of operative champions in the field or as integrators - persons in leading positions who work with integration, cooperation and establishment [28]. Since the 1980s, these core members have initiated health-promotion activities and driven them forwards, at the outset principally in the form of ad-hoc measures at departmental level in a decentralized and sectorally divided administration. This kind of engagement, combined with a position of power based on professional knowledge, has been shown to be of importance in health-development processes [29].

By virtue of the Swedish municipalities' increased degree of self-governance during the 1990s, with augmented areas of responsibility, their opportunities themselves to choose objectives and directions for their operations also increased. The turn of the century, in conjunction with reorganizations in three of our municipalities (A, B, C), saw the start of a trend towards more centralized goal-oriented local direction and control within the municipalitis.

The perceived lack of clarity among the municipalities under study on the issue of responsibility for local health promotion and the implementation of external goals (such as those in the PHP, as stipulated by the Swedish parliament in 2003) has only affected local developments to a limited extent. The municipalities, by taking their point of departure in their own needs and resources in the first instance, have themselves chosen the objectives and directions of their own health-promotion activities. Some safety work, which would conventionally be regarded as health promotion was, in several cases, not defined as local health promotion, but rather as an expression of a desire to offer municipal residents a safe community to grow up and live in.

All the municipalities (A-D) have established their health-promotion organization and development by both conscious and instinctive means and/or actions. A supportive factor in such development has been the presence of a strong and stable political majority, with a generally clear leadership, which was the case in municipalities A and C. In these municipalities, officials at departmental level perceived greater clarity in municipality-wide goals, which in turn facilitated intersectoral collaboration.

The municipalities' strained financial situation in the early 1990s and the shift back to more centralized governance in the municipalities seemed to be factors promoting the increased development of intersectoral collaboration and alliances within the municipal organization, and also with private and civic actors in the local arena. Different kinds of collaboration for health promotion have been developed, including networking and alliance formation. The view was expressed that this not only provided for efficiency and coordination gains, but also promoted public participation and influence. These enhancing factors, which facilitated action and implementation, and also increased participation and a sense of shared responsibility and ownership, have also been reported by Dressendorfer et al [30], Heward et al [31], Kegler et al [32], and Riley et al [33]. In their conceptual model of community capacity development, Dressendorfer et al [30] stress three facilitating factors to consider in the successful implementation and maintenance of health-promoting initiatives: well-functioning and engaging leadership, policy-making, and an existing operational infrastructure. Further, the authors regard community-level capacity building as the foundation for sustainable and long-term development, where support for development is provided by a combination of dedication, resources and competences. These factors correspond to the influences found in the current study.

Beyond these explicit external and internal influencing factors, the initiation of targeted local health-promotion actions showed a latent implicit connection with external health targets. One external influencing factor was the parallel national preparatory work for the national PHP, which started in 1995. Issues of public health were reported in the national and local media, and also revealed in local health statistics. Yanovitzky [34] has declared that, even if there is no clear evidence of a connection between media reporting and healthier behaviour, the policy-making of official authorities is affected, which has a positive influence on health promotion in the community.

The results of the three phases of development described in this study (initiation, action, achievements) also correspond to the three streams (problem, politics, policy) in the Kingdon model [12]. In all four municipalities, youths' alcohol habits were a known and defined problem. There was also a united political will to solve the problem. In Municipality D, for example, a renewal of alcohol policy was seen as one solution. And there were both officials and politicians who could be identified as window-opening policy entrepreneurs, and who made efforts to reach a broad and intersectoral foundation for the work. In Municipality D, the policy process led to a new alcohol policy, which was adopted and implemented. Another example is the policy process involved in the child impact analyses pursued in Municipality B.

The processes and factors related to health-promotion development in four Swedish municipalities can also be discussed from the perspective of theories of social change. Thompson and Kinne [11] describe a holistic view, where individuals are seen as actors in the context of a community system. There are four levels from a top-down perspective, starting with the external environment, and then descending in turn to the community, organization and individual. In this context, there are several connections with the development process of initiation, action and achievement that we have described. Even though the municipalities did not have any clearly defined or organized local health-promotion objectives, they did manage to develop measures and programs aimed at a healthier community.

This study's results correspond to those of Baum et al [35], in which successful health promotion is seen in terms of its sustainability. Several of the essential factors referred to by Baum and colleagues also emerged in this study. An important one is the ability to transform projects into well-integrated and long-term programmes. Others are a clearly defined leadership, adjustment to local preconditions, wide-ranging local support, intersectoral working, and an ability to tackle competing interests.

The weakest connection is the absence of a clearly expressed vision of health in the four municipalities. The municipalities' own conditions and needs have had an obvious influence on the development of long-term and intersectoral local health promotion. This is regarded as a key issue in health development by Dahlgren and Whitehead [36]. Further, that individuals and societies have the will to do something, if feasible, is a more important factor than what can be done technically.

The components presented and the increasing awareness of community health as a resource and important factor in the development of a well-functioning municipality, and also concern for a sustainable environment and economy, have led to increasing attention being paid to public-health issues and health promotion.

Studying local health promotion and its growth inductively and from a communal bottom-up perspective faces several difficulties. Each case is complex, contextualized, and not possible to control.

The municipalities in this study were not selected on the basis of how their local health-promotion activities were organized or developed. The purposeful sample was based on general factors, such as demography or geographical location; only thereafter was an investigation conducted into the municipalities' local health-promotion content, organization and development.

The aim of the study was exploratively to identify the municipalities' local health promotion from their own perspectives, and thereafter the factors that have influenced and characterized their development. In this context, the case-study approach is a suitable research strategy, since cases offer the opportunity for the study of processes and connections, in each particular case, and also a systematic way of handling and analyzing data [22,37]. We chose the inductive approach in order to capture the municipalities' views in an unprejudiced manner. We rejected a theory-driven deductive study design and analysis for the same reason; it would not have given us the municipalities' own objective views on their local health promotion. But, as Yin [22] puts it, an explorative study must have some stated purpose even though it may not present specific propositions.

Rootman's [26] seven principles for health promotion were taken as our starting point. Also, the design of this study, which encompassed a case-study protocol and several data-collection methods and data sources, enabled triangulation; validity is thereby strengthened through the interpretations of reasonableness that can be made from the different parts of the results.

The processes involved in the development of local health promotion could therefore be followed horizontally over time, and vertically, by monitoring, inter alia, decision-making processes, and also by the strategic sampling of interviewees at different levels in the municipal organizations and the examination of policy documents.

A common criticism of case studies is that, by virtue of their focus on a strictly delimited area, they hinder or prevent transformability. According to Yin [22], this criticism can be counteracted through the use of multiple cases. More cases make for a more robust study, in that analytic conclusions can be drawn from independent cases, which thereby strengthen their credibility. The number of inhabitants in Sweden's 290 municipalities ranges between 2 500 and 810 000. In relation to population size, the four municipalities in this study belong to the most common group of Swedish municipalities. There are no Swedish studies showing that bigger municipalities give greater priority to health promotion than smaller ones. But differences related to size have been found with regard to how municipalities have organized their local health promotion [38]. In order to promote population health and reach national and global goals for public health, intersectoral and multi-level cooperation and action are important. The municipality's role in this work as a local-level public sector actor has been stressed in, for example, the Ottawa Charter [1] and the Commission on Social Determinants of Health [39].

## Conclusions

Since the 1980s up to 2006, local health promotion has been developed in Sweden; in the four studied municipalities, there has been a growing awareness of public health as a structural issue and a sharper focus on different determinants of health. It is primarily local events, conditions and needs that have influenced the content, organization and development of local health promotion. The strategies that have principally been adopted represent various types of intersectoral collaboration, both internally within the municipal organization and externally with actors in the surrounding society. Local enthusiasts or champions, clear municipality-wide goals and leadership have been of greater importance than external influencing factors. Stable political will and direction, combined with clear and well-rooted goals and structures in the municipal organization, are preconditions for the sustainable development of long-term, coordinated and local health promotion.

## Competing interests

The authors declare that they have no competing interests.

## Authors' contributions

EJ: study design, data collection and analysis, manuscript preparation. PT: study design, data analysis and manuscript preparation. Both authors read and approved the final manuscript.

## Pre-publication history

The pre-publication history for this paper can be accessed here:

http://www.biomedcentral.com/1471-2458/10/455/prepub
